# High Sensitivity Cardiac Troponin T Versus Cardiac Troponin I on Prediction of Significant Coronary Artery Disease in Patients Hospitalized Due to Symptomatic Atrial Fibrillation

**DOI:** 10.3390/jcm14061855

**Published:** 2025-03-10

**Authors:** Tanja Thomsen, Maximilian Funken, Georg Nickenig, Marc Ulrich Becher

**Affiliations:** 1 Department of Medicine II, Heart Center Bonn, University Hospital Bonn, 53127 Bonn, Germany; maximilian.funken@ukbonn.de (M.F.); becher.marc@klinikumsolingen.de (M.U.B.); 2 Department of Medicine II, Städtisches Klinikum Solingen, 42653 Solingen, Germany

**Keywords:** atrial fibrillation, high-sensitivity cardiac troponin T, cardiac troponin I, coronary artery disease

## Abstract

**Background/Objectives**: Patients with atrial fibrillation (AF) often have symptoms and risk factors similar to those of patients with coronary artery disease (CAD). However, the clinical criteria for identifying AF patients who would benefit from coronary angiography (CA) remain vague. We evaluated the predictive value of cardiac troponin I (cTnI), high-sensitivity cardiac troponin T (hs-cTnT), and various clinical parameters for detecting significant coronary artery stenosis. **Methods**: We retrospectively analyzed symptomatic AF patients admitted to the University Hospital Bonn emergency department between 2015 and 2019 undergoing CA. Out of 183 AF patients, 93 were screened with cTnI and 90 with hs-cTnT. **Results**: A total of 47 out of 183 (26%) AF patients were diagnosed with significant coronary artery stenosis. The sensitivity for detecting CAD requiring intervention was 62.5% [95% CI, 40.6–81.2%] for cTnI and 100% [95% CI, 85.2–100%] for hs-cTnT. Median hs-cTnT concentrations were significantly higher in the “Revascularization-group” than in the “Non-Revascularization-group” (30.05 ng/L [95% CI, 26.5–54.8 ng/L], 23 patients vs. 15.3 ng/L [95% CI, 12.7–22.5 ng/L], 67 patients, *p* < 0.001). The calculated regression model that includes age, history of CAD, and hs-cTnT showed the best pretest performance with an AUC of 0.83, *p* = 0.008. Poor performance was observed for cTnI (AUC of 0.63, *p* = 0.098). **Conclusions**: This study demonstrates that the hs-cTnT assay is superior to the contemporary cTnI assay in predicting significant CAD requiring revascularization in patients hospitalized with AF. Older age, pre-existing CAD, impaired renal function, and a higher hs-cTnT cut-off showed the highest pretest probability of relevant CAD in patients hospitalized for AF.

## 1. Introduction

Atrial fibrillation (AF), the most common cardiac arrhythmia, is a frequent reason for emergency department (ED) admission [1]. Distinguishing between AF symptoms alone and those of concomitant ischemic heart disease is challenging. Patients with AF often complain of similar symptoms and have similar risk factors to those with coronary artery disease (CAD). Palpitations, chest pain [2], or angina pectoris [3,4] are common. The high pretest probability of shared risk factors, such as arterial hypertension, obesity, and age [5], further complicates the decision to perform additional testing for suspected concomitant myocardial ischemia and CAD. Furthermore, AF is a complication of acute myocardial infarction (MI) with an incidence of 10.4%, and patients with AF exhibit a higher incidence of severe CAD than patients without AF [6], suggesting that these patients may benefit from early coronary angiography (CA) with revascularization. Additionally, higher mortality rates have been observed in patients who developed AF in the setting of acute MI [7]. However, in previous studies, the incidence of AF in patients with CAD was found to be exceedingly low at just 0.08–5% [7,8,9,10,11,12]. In these cases, an association with MI and a severe impairment of left ventricular function was observed [8]. Conversely, several studies report a higher incidence of CAD in patients with AF, ranging among 16.6% [2], 34% [4], 36.3% [3], and 38% [13]. In addition to an electrocardiogram (ECG), echocardiography, and clinical examination, troponin is used as a marker of myocardial ischemia [14], with at least one value above the 99th percentile of the upper reference limit (URL) required to diagnose myocardial injury or infarction. Since both AF and ischemic myocardial injury can lead to troponin elevation [14,15], assessing patients with symptomatic AF is particularly relevant in light of the recently developed high-sensitivity assays for cardiac troponin (hs-cTn), which allow for a more precise measurement of concentrations than conventional assays [16]. These new high-sensitivity troponin assays are more sensitive for ischemic myocardial injury [14], and studies [17] confirm the added value of hs-cTn in the diagnosis of non-ST-segment elevation myocardial infarction (NSTEMI) in patients with AF compared to those without AF, demonstrating strong diagnostic performance. The use of diagnostic cut-offs that differ from the 99th percentile has been examined in the context of contemporary troponin I (cTnI) in AF and acute myocardial infarction, with findings suggesting improved specificity and positive predictive value [18]. Patients with elevated troponin levels are known to be a high-risk population and elevated hs-cTnT levels are strongly associated with higher mortality in patients with AF [19,20]. The aim of this study was to assess whether the transition from cTnI to hs-cTnT has led to disparities in identifying AF patients who benefit from CA and to identify additional factors that may predict the need for revascularization among AF patients.

## 2. Materials and Methods

### 2.1. Data Collection

Hospitalized patients diagnosed with AF between 1 January 2015 and 31 December 2019 who underwent CA were identified using the International Classification of Diseases 10th revision (ICD-10) code I48 (Atrial fibrillation and flutter, Table 1) and the German procedure classification version 2019 (OPS19: 1-275 Transarterial left heart catheterization) from medical accounting reports and reviewed in ORBIS^®^ (Dedalus HealthCare, Bonn, Germany, ORBIS 08044207.03000.DACHL), the clinical health software system of the University Hospital Bonn (UKB). ED records, medical and nursing reports, echocardiography and angiography reports, and laboratory information were reviewed. If relevant records, such as ECG interpretation, were not documented, these patients were excluded. All medical information records were reviewed manually.

### 2.2. Selection Criteria

Patients with a diagnosis of symptomatic new-onset or recurrent AF in the ED of the UKB without hospital transfer were included. Patients were excluded if AF was detected later than at the time of admission, if it was detected incidentally during a routine testing or during monitoring, or if it was detected after admission in patients whose hospitalization was due to another medical condition and treatment.

### 2.3. Patient Data

Data on patient demographics (age, sex, history of AF or CAD, hypertension, diabetes mellitus type II, smoking, family history of cardiovascular events, and obstructive sleep apnea) and clinical presentation (blood pressure, heart rate, tachyarrhythmia, AF recurrence, electro cardioversion attempt, angina pectoris [CCS score [21], if available], palpitations, dyspnea [EHRA score [22], if available], cardiac decompensation, and (pre-) syncope were included. If clinical history and parameters were not provided, the information was considered negative. Tachyarrhythmia was defined as a heart rate greater than 100 beats per minute, taking into account the first or highest value recorded on admission to the ED. Angina pectoris symptoms were considered positive if the patient reported chest pain, chest pressure, or other chest discomfort. Cardiac decompensation was defined by the presence of clinical manifestations provided in the clinicians’ reports, including oedema, pleural effusion, ascites, or pulmonary venous congestion.

Data collected from the echocardiographic records were ejection fraction (EF), hypokinesia, right heart dysfunction, pulmonary hypertension, left atrial size, and mitral valve regurgitation. The global and regional myocardial contraction patterns were interpreted through the application of semiquantitative or visual assessment. The varying reporting terminology of the echocardiography reports was analyzed by defining them into uniform parameters and numbers (Table A1). If the EF value was not provided in the ED protocol, we considered the values obtained from inpatient echocardiography and levocardiography. If mitral regurgitation was not detected by inpatient transthoracic echocardiography, data from transesophageal echocardiography were used. If a right heart catheterization was also performed, pulmonary hypertension data were used.

Angiographic data included the presence of CAD, the number of vessels involved, and revascularization attempts. The definition of relevant CAD is provided as an indication for revascularisation by percutaneous coronary intervention (PCI) or coronary artery bypass graft (CABG) in the angiography report, on the basis of a relevant coronary artery stenosis, as determined by the investigator. Laboratory data included cTnI levels on admission and three hours later, following the (guideline-based) 0 h/3 h algorithm, as well as hs-cTnT levels at admission and one hour later, following the (guideline-based) 0 h/1 h algorithm. The initial and subsequent troponin measurements were considered. The concentrations of lactate, potassium, glucose, hemoglobin, and the glomerular filtration rate (GFR), according to the MDRD (modification of diet in renal disease) formula by the central laboratory of the UKB upon admission and LDL and HbA1c levels during hospitalization, were also recorded.

### 2.4. Assay Data

The contemporary sensitive cTnI Dimension Vista Assay from Siemens Healthineers (Siemens Healthcare GmbH, Erlangen, Germany) was used at the UKB until 21 August 2017 and has a 99th percentile of 45 ng/L (0.05 ng/mL). The analytical imprecision of the assay, calculated as the coefficient of variation (CV) less than or equal to 10%, has a concentration of 0.04 ng/mL at the 99th percentile. The lowest measurable concentration, the “limit of blank” (LoB), formerly called the limit of detection (LoD), is 15 ng/L (0.02 ng/mL). Since then, the UKB has been using the hs-cTnT assay from Roche Diagnostics (Roche Diagnostics International AG, Rotkreuz, Switzerland) with a 99th percentile of 14 ng/L, a LoD of 3 ng/L, and a lowest concentration of 13 ng/L with a CV of less than or equal to 10% at the 99th percentile.

A total of 93 patients were screened with cTnI (from 1 January 2015 to 21 August 2017), while 90 patients were screened with hs-cTnT (transition date to 31 December 2019). 

Upper reference limits and thresholds for the respective assay characteristics are available from the manufacturers, in the literature [23], and on the website of The International Federation of Clinical Chemistry and Laboratory Medicine (IFCC) [24]. Hs-cTn assays differ from contemporary cardiac troponin assays in their ability to measure troponin concentrations above the LoD in 50% of a healthy population and in having a lower CV (less than or equal to 10% at the 99th percentile vs. 10% to 20% for contemporary assays [14]).

### 2.5. Statistical Analysis

Baseline information is presented as medians and interquartile ranges for metric variables and as observed number percentages for categorical variables. Metric variables are shown as continuous and binary variables based on their clinical reference limits. In addition, some parameters were grouped for better statistical analysis. Upper limits of troponin assays were defined as the 99th percentile URL for each assay.

In addition, for hs-cTnT, we employed the 0 h/1 h rule-out and rule-in guideline algorithm for suspected non-ST-segment elevation acute coronary syndrome (NSTE-ACS) [25], as this is a crucial differential diagnosis for symptomatic AF patients in the ED. This application is explained by the assay-specific intervals of troponin measurements to verify a kinetic pattern and to “rule in” or “rule out” patients for discharge, admission to a coronary care unit, further diagnostics, and therapeutic intervention [25].

The indication for revascularization in the angiography report was used to assign patients into “Revascularization” (R-Group) and “Non-Revascularization” (Non-R-Group). Depending on the type and sample size of the given data, the Chi-squared test or Fisher’s exact test for binary variables and the Mann–Whitney U test for categorical and metric variables were used to distinguish differences between these two groups. Promising predictors from this first analysis were tested and estimated using a binary logistic regression model to be valid risk factors for predicting intervention (= R-Group). To reduce the likelihood of the model being overly influenced by random outliers (overfitting) [26,27], we limited our regression analyses to a maximum of three predictor variables. The results of the binary logistic regression models mentioned above were compared with exact logistic regression to examine the best fit for small sample sizes. Furthermore, 1000-sample bootstrapping was performed to validate the final model in terms of data robustness. Model performance was determined by the receiver-operating characteristic (ROC) curve and the area under the curve (AUC). Moreover, model performances were compared by testing the AUCs with *t*-tests to expose statistically significant differences and advantages of the models. Since ROC curves can be considered as normally distributed, we used the given value of the AUC as the mean and the given standard error of the model to calculate the standard deviation. Statistical analyses were performed with IBM SPSS Statistics (IBM Corp. Released 2021. IBM SPSS Statistics for Windows, Version 28.0. IBM Corp., Armonk, NY, USA) and STATA 15.1 (StataCorp. 2017. Stata Statistical Software: Release 15. StataCorp LLC, College Station, TX, USA).

## 3. Results

### 3.1. Study Population

**Figure 1:**
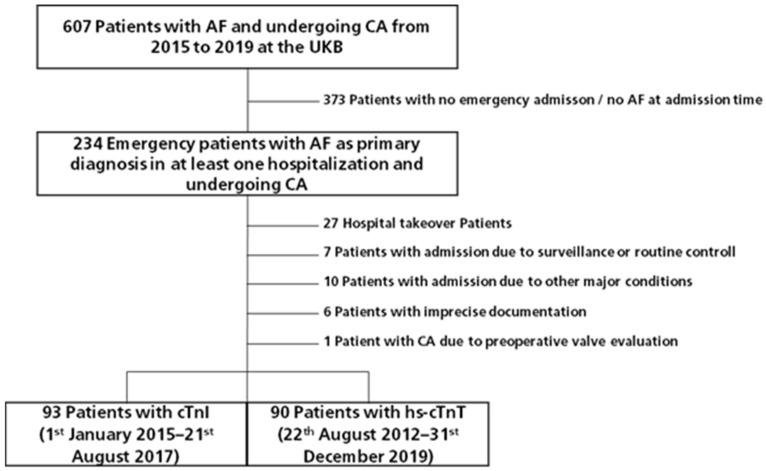
Patient flow chart. AF: Atrial fibrillation; UKB: University Hospital Bonn; CA: coronary angiography; CAD: coronary artery disease; cTnI: cardiac troponin I; hs-cTnT: high sensitivity cardiac troponin T.

From 1 January 2015 to 31 December 2019, 607 patients with coded AF underwent CA (Figure 1). A total of 373 patients dropped out due to either a lack of admission to the ED or the absence of AF at the time of admission. A total of 27 patients were hospital takeovers. Patients were excluded if they were admitted for symptoms other than AF, if AF was incidentally detected in patients undergoing a routine checkup or monitoring, and if CA was performed for preoperative reasons. Six patients were excluded because of imprecise documentation.

### 3.2. Baseline Characteristics

In consideration of the transition of troponin assays at UKB in August 2017, 93 out of 183 patients (51%) were tested with cTnI, while 90 patients (49%) were tested with hs-cTnT, exhibiting a similar distribution of the assay groups. The baseline information of the patients for each assay (Table 2) indicates no statistically significant differences between the groups, with the exception of a slightly higher prevalence of arterial hypertension hs-cTnT-group.

Baseline information for the R-Group and Non-R-Group are presented in Table 3. Age and prior diagnosis of CAD differed statistically significantly between those R- and Non-R-Groups, with older age (median of 77 years vs. 74 years, *p* = 0.012) and a higher prevalence of previously diagnosed CAD in the R-Group (51.1% vs. 29.4% in the Non-R-Group, *p* = 0.007). Systolic and diastolic blood pressure were statistically significantly higher in the R-Group (*p* = 0.008, *p* = 0.035), while gender, tachyarrhythmia, and chest pain did not show statistically significant differences within the groups. However, a statistically significant variation between the groups was observed for mitral valve regurgitation (MR) (*p* = 0.014), but not for attempts of cardioversion, AF recurrence, hypokinesia, EF, or the size of the left atrium (Table 4).

Of the 183 AF patients, 47 (26%) were diagnosed with CAD and required revascularization, as detailed in Table 5. Of the remaining 136 patients (74%), no relevant stenosis was observed, with a general prevalence of CAD of 47.1%. However, the sensitivity for the presence of CAD requiring intervention (=R-Group) was only 62.5% (95% CI, 40.6–81.2%) for cTnI (using the 99th percentile cut-off) and, in contrast to that, 100% (95% CI, 85.2–100%) for hs-cTnT (using the 99th percentile cut-off). Information and values of the troponin assays and algorithms can be found in Table 6. Additional laboratory findings showed that impaired renal function (GFR < 60 mL/min) was observed to be more prevalent in the R-Group, whereas NT-proBNP and lactate did not differ significantly between the groups (Table 7).

### 3.3. Cardiac Troponin Assays

**Figure 2:**
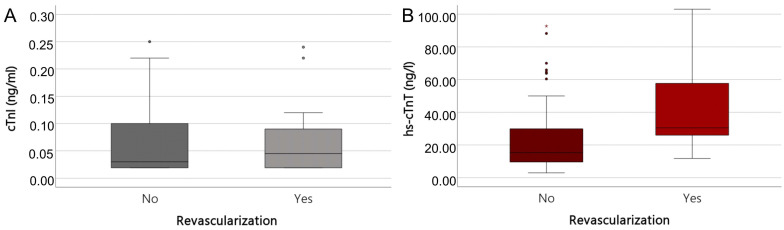
Boxplot of cTnI (**A**) in ng/mL and hs-cTnT in ng/L (**B**). Median cTnI 0.03 ng/mL (No Revascularization) and 0.045 ng/mL (Revascularization) (**A**); Median hs-cTnT 15.3 ng/L (No Revascularization) and 30.5 ng/L (Revascularization) (**B**). Extreme value outliers (defined as being greater than three times the interquartile range) are indicated as asterisk.

Troponin levels are presented in three different formats: as metrics, as binary variables (using the URL), and as categorized according to the NSTE-ACS guideline algorithm. The rule-out cut-off for hs-cTnT Elecsys by Roche is 12 ng/L, while a new rule-out diagnostic cut-off level of 22 ng/L has also been implemented. This was based on a combination of statistical evaluation of different cut-offs using ROC analyses and the clinical context, given that the median of our study population was 21.9 ng/L and even in the Non-R-Group, the hs-cTnT median was above the 99th percentile URL. The median hs-cTnT concentration (Figure 2B) was significantly higher in the R-Group (30.5 ng/L, 95% CI, 26.5–54.8 ng/L) than in the Non-R-Group (15.3 ng/L, 95% CI, 12.7–22.5 ng/L), *p* < 0.001. This suggests that for the specific cohort of patients presenting AF-related symptoms in the ED, a higher cut-off value may be advantageous in identifying relevant stenoses resulting from concomitant myocardial ischemia associated with CAD.

However, the sensitivity for the presence of CAD requiring intervention (= R-Group) was only 62.5% (95% CI, 40.6–81.2%) for cTnI (using the 99th percentile cut-off) and, in contrast to that, 100% (95% CI, 85.2–100%) for hs-cTnT (using the 99th percentile cut-off) (Table 8).

### 3.4. Predictors for a Significant Coronary Obstruction in AF Patients

**Figure 3:**
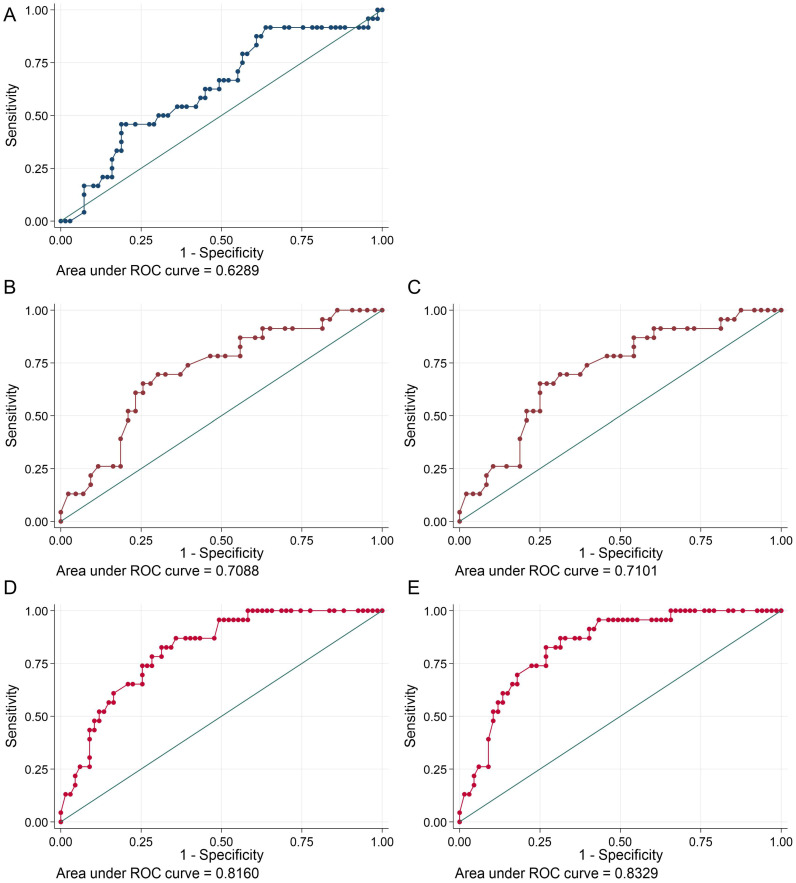
ROC curves and AUC. cTnI (**A**) (blue curve), hs-cTnT with 99th percentile reference limit (**B**) and NSTE-ACS guideline algorithm (**C**) (brown curves), hs-cTnT with 22 ng/L as reference limit (**D**), and rule-out cut-off level at 22 ng/L (**E**) (red curves).

The objective was to ascertain which parameters can be identified as independent risk factors for the prediction of relevant stenoses in patients with AF. This was accomplished using calculated regression models (Figure 3), including age, history of CAD, and troponin levels/cut-offs. The cTnI model exhibited a poor performance (AUC of 0.63, *p* = 0.098) (Figure 3A). In comparison, the hs-cTnT model (whether utilizing the 99th percentile or rule-in/rule-out algorithm) demonstrated a statistically significant improvement in performance relative to the cTnI model. Considering that the results of our prediction models with a cut-off at 22 ng/L (AUC = 0.83) (Figure 3D and E) were preferable to the models incorporating the 99th percentile URL (Figure 3B) or guideline algorithm (AUC = 0.71) (Figure 3C), we employed the *t*-test to identify statistically significant differences between these models, which indicated superior performance for the new cut-off value (*p* = 0.0554).

## 4. Discussion

The distinction of symptoms resulting from AF alone or in conjunction with coronary artery stenosis is a daily challenge in the ED. In addition to their clinical manifestations, patients with AF and CAD often share similar risk factors, including advanced age and arterial hypertension [3,5,21]. Consequently, we sought to investigate whether switching to an hs-cTn assay could improve the ability to distinguish between symptomatic AF patients with significant CAD and those without relevant stenosis. To achieve this objective, we utilized retrospective data collected around the assay transition date (21 August 2017) when the non-high-sensitive cTnI was replaced by the hs-cTnT assay. The results demonstrated that the hs-cTnT assay exhibited superior predictive capacity for significant CAD in emergently hospitalized AF patients compared to the previously used cTnI assay. Notably, this superiority was most pronounced at a higher threshold of 22 ng/L, as opposed to the conventional cut-off of 12 ng/L. Furthermore, besides elevated hs-cTnT, other factors, including advanced age, a history of CAD, and impaired renal function, were identified as the strongest predictors of significant CAD in this cohort of symptomatic AF patients.

Previous studies extensively compared AF patients with non-AF patients to identify clinical risk factors and outcomes [5,6,7,8,12]; for instance, it has been demonstrated that AF patients with CAD tend to have greater disease severity, and those with AF and MI have a significantly higher number of diseased coronary arteries [28]. In the GUSTO-I trial, AF patients had a higher prevalence of three-vessel disease, lower arterial reperfusion rates, and lower EF than those without AF [6]. Similarly, higher mortality rates were observed in AF patients who developed AF in the setting of acute MI (The GUSTO-III experience [7]). These findings suggest that early CA and PCI should be strongly considered in AF patients [6]. However, further data are needed on prognostic factors associated with severe CAD and coronary obstruction in this population. Our study provides additional information on clinical symptoms, echocardiographic, and angiographic findings, particularly in symptomatic AF patients presenting to the ED. We investigated the role of both conventional cTnI and hs-cTnT, as well as other predictors of significant coronary stenosis. The new high-sensitivity assays are known to be superior to the contemporary assays due to improved sensitivity [29] and better differentiation between normal and mildly elevated values [25]. These assays are more sensitive for ischemic myocardial injuries [14] and Sörensen et al. confirmed the usefulness of hs-cTnI in the diagnosis of NSTEMI in AF patients compared to those without AF, demonstrating significant diagnostic benefits [17]. Given that AF is associated with elevated cardiac troponin levels—and that elevated troponin levels independently predict an increased risk of all-cause mortality and cardiovascular events [30]—better diagnostic strategies for identifying AF patients in the ED who may benefit from early invasive CA are urgently needed. cTnI has been described as having a limited positive predictive value for a significant coronary obstruction [31] in the acute setting in patients with AF. Only 26% of patients with elevated cTnI had significant stenosis, while 33% of those with normal cTnI still exhibited significant stenosis on CA [31], which is consistent with our study results. Moreover, using AF-specific cut-offs, rather than the standard 99th percentile, has been shown to improve the positive predictive value of conventional troponin assay in AF patients with suspected MI [18].

Following the widespread implementation of high-sensitivity assays in clinical practice, a broader spectrum of myocardial damage has become detectable, highlighting the need for a more nuanced diagnostic approach. Consequently, clinicians must adopt a stratified approach to accurately identify patients at risk.

Our study introduces an AF-specific hs-cTnT cut-off approach with potential prognostic relevance. In the Non-R-Group, where the prevalence of CAD was 47.1%, the median hs-cTnT was 15.3 ng/L. In comparison, a previous study of AF patients with known CAD reported a median of hs-cTnT of 12.6 ng/L (vs. 8.7 ng/L in non-AF patients with CAD), along with significantly reduced GFR (<60 mL/min) in the AF group [32]. Similarly, a statistically significant reduction in GFR was observed in AF patients with suspected NSTEMI compared with non-AF patients [17]. Whether this association with impaired renal function is primarily driven by older age in AF patients or reflects an independent pathophysiological mechanism remains unclear. Further research is needed to clarify these relationships.

The well-established link between mitral valve regurgitation [8,9,11] and AF has also been reinforced by our findings, which suggest an additional association between these echocardiographic features and significant coronary obstruction.

A recent German study by K.M. Stoyanov et al. included patients with AF admitted to the ED at Heidelberg University Hospital over four years to assess the relationship between hs-cTnT and mortality [19]. The median hs-cTnT level in that study was 17.0 ng/L [19]. In contrast, our cohort exhibited higher hs-cTnT values (median hs-cTnT of 21.9 ng/L), likely due to different inclusion criteria. While the previous study focused on overall mortality in unselected AF patients, our study specifically analyzed symptomatic AF patients undergoing CA, with the primary endpoint being the need for revascularization. Nevertheless, studies confirm that elevated hs-cTnT levels are strongly associated with increased mortality in AF patients [19,20].

The increasing use of hs-cTn necessitates corresponding adjustments in clinical decision-making. Risk models that integrate multiple predictive factors—such as renal function and specific troponin cut-offs—could improve the selection of AF patients who would benefit most from CA. The insights gained from our study could inform future research aimed at refining diagnostic strategies and optimizing early intervention for AF patients at high risk of significant CAD.

The limitations of this study must be acknowledged. First, as a retrospective, single-center, and observational study, the findings may not be generalizable. The study was conducted in a single ED of a tertiary care hospital and is based on a single dataset. Future research should validate these results using larger, multicenter cohorts. Additionally, the accuracy of patient assessment may be influenced by the experience of attending physicians and the quality of medical documentation, introducing potential subjective biases. Finally, further prospective studies are needed to refine the proposed clinical prediction model and investigate the link between impaired renal function and troponin elevation in AF patients, as well as other clinical parameters that may be relevant in acute CAD risk assessment in this population.

## 5. Conclusions

Here we demonstrate that the hs-cTnT assay is superior to the cTnI assay in predicting significant CAD in patients hospitalized emergently with symptomatic AF. Based on the contemporary troponin assay, the sensitivity for the presence of CAD requiring intervention was only 62.5% [95% CI, 40.6–81.2%] for cTnI, compared to 100% (95% CI, 85.2–100%) for hs-cTnT. Moreover, we identified predictors (older age, previous history of CAD, and impaired renal function), including different cut-off levels of hs-cTnT, with the most promising being the higher cut-off level in patients with AF. This encourages future prospective studies to ameliorate the diagnosis of AF patients with likely relevant coronary stenosis by employing additional clinical diagnostic factors and hs-cTn assay thresholds.

## Figures and Tables

**Table 1:** ICD-10-German Modification.

I48.0 Paroxysmal atrial fibrillation
I48.1 Persistent atrial fibrillation
I48.2 Permanent atrial fibrillation
I48.9 Unspecified atrial fibrillation and atrial flutter

**Table 2:** Baseline characteristics assay groups.

Baseline Characteristics	n	cTnI, n = 93 (51%)	%/ (Quartiles)	hs-cTnT, n = 90 (49%)	%/ (Quartiles)	*p* Value
Age (years)	183	74	(65–80)	75	(66–80)	0.515
Male	183	53	57.0	42	46.7	0.620
Female		40	43.0	48	53.3	
First diagnosis of AF	183	41	44.1	41	45.6	0.842
Prior CAD diagnosis	183	31	33.3	33	36.7	0.637
Family history	183	15	16.1	8	8.9	0.141
Diabetes mellitus	183	18	19.4	24	26.7	0.241
Smoking	183	18	19.4	16	17.8	0.784
Former Smoking	183	26	28.0	4	4.4	0.845
Hypertension	183	72	77.4	80	88.9	0.039
OSA	183	7	7.5	8	8.9	0.738
Clinical parameters						
Systolic BP (mmHg)	128	139	(127–154)	135	(112–160)	0.682
Diastolic BP (mmHg)	126	82	(68–98)	85	(70–98)	0.866
Tachyarrhythmia	182	74	79.6	68	75.6	0.428
Heart rate (bpm)	182	128	(104–150)	120	(95–145)	0.113
Chest pain	183	33	35.5	29	32.2	0.642
Palpitations	183	39	41.9	28	31.1	0.130
Cardiac decompensation	183	22	23.7	27	30.0	0.334
Syncope	183	6	6.5	7	7.8	0.728
Presyncope	183	2	2.2	1	1.1	1.000
CA						
Indication for Revascularization	183	24	25.8	23	25.6	

AF: Atrial fibrillation; CAD: Coronary artery disease; OSA: Obstructive sleep apnoea; BP: Blood pressure; bpm: Beats per minute; CA: Coronary angiography.

**Table 3:** Baseline characteristics R-Group (left) and Non-R-Group (right).

Baseline Characteristics	n	R-Group, n = 47 (26%)	%/ (Quartiles)	Non-R-Group, n = 136 (74%)	%/ (Quartiles)	*p* Value
Age (years)	183	77	(71–82)	74	(65–79)	0.012
Male	183	27	57.4	74	54.4	0.719
Female	183	20	42.6	62	45.6	
First diagnosis of AF	183	21	44.7	61	44.9	0.984
Prior CAD diagonsis	183	24	51.1	40	29.4	0.007
Family history	183	6	12.8	17	12.5	0.962
Diabetes mellitus	183	12	25.5	30	22.1	0.626
Smoking	183	6	12.8	28	20.6	0.236
Former Smoking	183	11	23.4	39	28.7	0.486
Hypertension	183	39	83.0	113	83.1	0.986
OSA	183	4	8.5	11	8.1	1.000
Clinical parameters						
Systolic BP (mmHg)	128	150	(130–170)	134	(112–153)	0.008
Diastolic BP (mmHg)	126	93	(78–103)	81	(68–95)	0.035
Tachyarrhythmia	182	39	83.0	103	75.7	0.342
Heart rate (bpm)	182	130	(104–146)	125	(96–150)	0.498
Chest pain	183	19	40.4	43	31.6	0.273
Palpitations	183	17	36.2	50	36.8	0.942
Cardiac decompensation	183	16	34.0	33	24.3	0.193
Syncope	183	2	4.3	11	8.1	0.520
Presyncope	183	2	4.3	1	0.7	0.162

AF: Atrial fibrillation; CAD: Coronary artery disease; OSA: Obstructive sleep apnoea; BP: Blood pressure; bpm: Beats per minute; CA: Coronary angiography.

**Table 4:** ECV and Echocardiographic data.

Cardioversion	n	R-Group, n = 47 (26%)	%/ (Quartiles)	Non-R-Group, n = 136 (74%)	%/ (Quartiles)	*p* Value
ECV performed	183	21	44.7	69	50.7	0.475
Primary ECV success	183	17	36.2	62	45.6	0.262
ECV success	183	16	34.0	66	48.5	0.086
Spontaneous conversion	182	14	29.8	46	33.8	0.591
Pharmacological conversion	182	2	4.3	6	4.4	1.000
AF recurrence	180	4	8.5	22	16.2	0.200
**Echocardiography**						
Hypokinesia focal	183	7	14.9	18	13.2	0.776
Hypokinesia global	183	9	19.1	27	19.9	0.917
Hypokinesia in total	183	16	34.0	45	33.1	0.905
EF (%)	183	52	(40–58)	55	(40–60)	0.467
EF ≤ 35%	183	9	19.1	27	19.9	0.917
V. cava dilatation	183	5	10.6	12	8.8	0.772
Right Heart Dysfunction	183	32	68.1	75	55.1	0.122
LA size (ml)	165	70	(52–90)	60	(53–80)	0.258
LA dilatation	165	18	38.3	36	26.5	0.106
sPAP (mmHg)	100	35	(31–46)	36	(25–44)	0.199
sPAP (≥ 25 mmHg)	100	31	66.0	58	42.6	0.016
MR	182					0.014
0 (= 0)	15	2	4.3	13	9.6	
I (= 1)	72	14	29.8	58	42.6	
I–II (=1.5)	37	10	21.3	27	19.9	
II (=2)	43	15	31.9	28	20.6	
II+ (=2.222)	3	2	4.3	1	0.7	
II–III (=2.5)	8	3	6.4	5	3.7	
III (=3)	4	1	2.1	3	2.2	
MR classes						0.096
0 (0)	15	2	4.3	13	9.6	
1 (I, I–II, II)	152	39	83.0	113	83.1	
2 (II+, II–III, III)	15	6	12.8	9	6.6	
MR > II	182	6	12.8	9	6.6	0.220

ECV: Electrical cardioversion; AF: Atrial fibrillation; EF: Ejection fraction; V. cava: Vena cava; LA: Left atrium; sPAP: Systolic pulmonary artery pressure; MR: Mitral regurgitation.

**Table 5:** Coronary angiography report.

CA	R-Group, n = 47 (26%)	%/ (Quartiles)	Non-R-Group, n = 136 (74%)	%/ (Quartiles)	*p* Value
CAD diagnosis	47	100.0	64	47.1	<0.001
Vessels					<0.001
0	0	0.0	81	59.6	
1	11	23.4	22	16.2	
2	10	21.3	19	14.0	
3	26	55.3	14	10.3	

CA: Coronary angiography; CAD: Coronary artery disease.

**Table 6:** Troponin testing.

Troponin Assay	n	R-Group	%/ (Quartiles)	Non-R-Group	%/ (Quartiles)	*p* Value
cTnI	93	n = 24 (26%)		n = 69 (74%)		
cTnI (0 h)	93	0.045	(< 0.02–0.095)	0.03	(<0.02–0.1)	0.753
cTnI (>0.05 ng/mL)	93	10	41.7	29	42.0	0.975
cTnI (3 h)	75	0.12	(0.053–0.8)	0.08	(0.02–0.2)	0.202
cTnI 3 h (>0.05 ng/mL)	75	20	83.3	55	79.7	0.412
∆0–3 h cTnI	75	0.051	(0.003–0.293)	0.01	(0.001–0.08)	0.285
∆0–3 h cTnI time (h)	75	4.7	(2.9–8.2)	4,1	(2.7–7.7)	0.594
Elevated cTnI	93	15	62.5	36	52.2	0.477
hs-cTnT	90	n = 23 (26%)		n = 67 (74%)		
hs-cTnT (0 h)	90	30.5	(25.5–60.6)	15.3	(9.6–31.1)	<0.001
hs-cTnT (>14 ng/L)	90	22	95.7	37	55.2	<0.001
hs-cTnT (1 h)	71	33.7	(25.8–60.2)	26.3	(16.8–47.8)	0.050
hs-cTnT (1 h) (>14 ng/L)	71	22	95.7	41	61.2	0.261
∆0–1 h hs-cTnT	71	4.5	(3.9–8.2)	4.2	(0.6–10.7)	0.363
∆0–1 h hs-cTnT time (h)	71	1.3	(1.0–2.0)	1.4	(1.1–1.8)	0.936
Elevated hs-cTnT	90	23	100.0	43	64.2	<0.001
NSTE-ACS guideline algorithm						
hs-cTnT rule-in	90	13	56.5	23	34.3	0.062
hs-cTnT observe	90	10	43.5	25	37.3	0.603
hs-cTnT rule-out	90	0	0.0	19	28.4	0.002
hs-cTnT (rule-in + observe)	90	23	100.0	48	71.6	0.002
hs-TnT cut-off 22 ng/L						
hs-cTnT observe	90	9	39.1	12	17.9	0.039
hs-cTnT rule-out	90	1	4.3	32	47.8	<0.001
hs-cTnT (rule-in + observe)	90	22	95.7	35	52.2	<0.001

cTnI: cardiac troponin I; hs-cTnT: high sensitivity troponin T. Elevated cTnI was defined as value above the upper reference limit (0 h or 3 h value > 0.05 ng/mL). Elevated hs-cTnT was defined as value above the URL (0 h or 1 h value > 14 ng/L). NSTE-Guideline algorithm by ESC [25] for hs-cTnT by Elecsys Roche.

**Table 7:** Laboratory findings.

Laboratory	n	R-Group, n = 47 (26%)	%/ (Quartiles)	Non-R-Group, n = 136 (74%)	%/ (Quartiles)	*p* Value
NT-proBNP	19	8420	(935–19,929)	1708	(727–4003)	0.195
Lactate	170	1.93	(1.6–2.64)	1.91	(1.48–2.45)	0.433
Lactate (>1.8 mmol/L)	170	22	46.8	71	52.2	0.728
Glucose	168	132	(115–160.5)	125	(107–152)	0.175
Potassium	174	4.04	(3.7–4.61)	4.12	(3.76–4.43)	0.975
Potassium pathologic (3.6–4.8 mmol/L reference)	174	14	29.8	32	23.5	0.296
Hb	183	13	(12.2–14.6)	13.9	(12.5–14.9)	0.113
Hb (f < 12 g/dL, m < 14 g/dL)	183	22	46.8	47	34.6	0.136
GFR	183	57.42	(44.6–>70)	66.98	(52.66–>70)	0.016
GFR reduced (<60 mL/min)	183	26	55.3	43	31.6	0.004
LDL	96	94.5	(71.5–123.3)	100	(80.8–131.3)	0.507
LDL (>100 mg/dL)	96	12	25.5	33	24.3	0.365
HbA1c	26	6.1	(5.6–6.8)	6.2	(5.5–7)	0.787
HbA1c (>5.7%)	26	6	12.8	11	8.1	1.000

f: female; m: male; GFR: glomerular filtration rate.

**Table 8:** Sensitivity and specificity of troponin testing.

	cTnI	hs-TnT 99th Percentile (14 ng/L)	hs-TnT Cut-Off 22 ng/L
Sensitivity (%)	62.5	100	86.96
Specifity (%)	47.8	35.8	56.72
False Positive (%)	52.2	64.2	52.17
False Negative (%)	37.5	0	37.50
LR+	1.2	1.6	2
LR−	0.78	-	0.2

## Data Availability

The datasets used and analyzed during the current study are available from the corresponding author upon reasonable request.

## Appendix A

**Table A1:** Echocardiographic parameters.

Parameter	Terminology	Defined Values
Ejection fraction	“good”	60%
	“preserved”	55%
	“slightly reduced”	50%
	“moderately reduced”	45%
	“severely reduced”	35%
Mitral valve regurgitation	“inconspicuous”	0
		I
		II
	“high grade”	II+
		III
		IV
Right heart dysfunction	Reduced function of the right ventricle	TAPSE < 2 cm
	Pulmonary Hypertension	sPAP ≥ 25 mmHg
	Right Ventricle Dilatation	
Diameter V. cava	“not dilated”, “congested”, “normal”	Diameter < 2 cm
	“dilated”, “congested”	Diameter > 2 cm
LA volume	reference range	22–52 mL → 52 mL (f)
		18–58 mL → 58 mL (m)
	mildly abnormal	53–63 mL → 63 mL (f)
		59–68 mL → 68 mL (m)
	moderately abnormal	63–72 mL → 72 mL (f)
		69–78 mL → 78 mL (m)
	severely abnormal	≥73 mL → (f)
		≥79 mL → (m)

TAPSE: Tricuspid annular plane systolic excursion; sPaP: Systolic pulmonary artery pressure; LA: Left atrium; EF: Ejection fraction; V. cava: Vena cava; m: Male; f: Female. If EF, LA, or sPaP range was reported, the higher severity grade was selected. Mitral valve regurgitation ranges were transferred to continuous variables. LA volumes of Lang et al. [33].
